# Multiple evolutionary routes to immune diversification across animals

**DOI:** 10.1093/intimm/dxag018

**Published:** 2026-04-15

**Authors:** Mizuki Taguchi, Sébastien de La Forest Divonne, Ryo Morimoto

**Affiliations:** Department of Molecular Biology, Umeå University, Umeå 901 87, Sweden; The Laboratory for Molecular Infection Medicine, Sweden (MIMS), Umeå University, Umeå 901 87, Sweden; Umeå Centre for Microbial Research (UCMR), Umeå University, Umeå 901 87, Sweden; Department of Molecular Biology, Umeå University, Umeå 901 87, Sweden; The Laboratory for Molecular Infection Medicine, Sweden (MIMS), Umeå University, Umeå 901 87, Sweden; Umeå Centre for Microbial Research (UCMR), Umeå University, Umeå 901 87, Sweden; Department of Molecular Biology, Umeå University, Umeå 901 87, Sweden; The Laboratory for Molecular Infection Medicine, Sweden (MIMS), Umeå University, Umeå 901 87, Sweden; Umeå Centre for Microbial Research (UCMR), Umeå University, Umeå 901 87, Sweden

**Keywords:** antigen receptor, genome editing, hematopoiesis, invertebrates

## Abstract

Adaptive immunity is often viewed as a defining innovation of vertebrates, characterized by somatically diversified antigen receptors and clonal lymphocyte lineages. Yet the evolutionary origins of such systems remain incompletely understood. In this review, we examine adaptive immunity from a comparative perspective across Metazoa, focusing on the design principles that link molecular diversification, immune cell differentiation, and proliferative dynamics. We first outline the two adaptive immune architectures found in vertebrates. Jawed vertebrates employ immunoglobulin-based and T-cell receptor-based recognition generated through recombination-activating gene (RAG)-mediated V(D)J recombination, whereas jawless vertebrates assemble variable lymphocyte receptors using cytidine-deaminase-dependent diversification of leucine-rich repeat modules. Despite their distinct molecular entities, these systems converge on shared design principles, including somatic diversification, developmental restriction of genome editing, immune cell differentiation, and specialized microenvironments for immune education. To introduce evolutionarily more ancient systems, several diversification mechanisms of antigen receptors in invertebrates will be subsequently surveyed. These systems generate substantial molecular diversity without canonical clonal selection, suggesting that immune recognition and diversification can be achieved through multiple evolutionary strategies. Particular attention is given to emerging insights into invertebrate immune cell diversification, where single-cell transcriptomics is revealing complex hematopoietic lineages and regulatory programs. These observations suggest that adaptive immunity did not emerge abruptly but rather represents one solution within a broader evolutionary landscape of immune diversification strategies. Understanding how diversification, proliferation, and cellular organization interact across animal lineages will help clarify the fundamental design constraints that shaped the evolution of vertebrate adaptive immune systems.

## Teaser

Immune diversification strategies in Metazoa

## Introduction: evolutionary emergence of adaptive immunity

Immune systems can be described as information-processing solutions to a universal biological problem: how to detect danger while preserving self. All organisms must solve this problem, yet they do so using remarkably different molecular architectures. This diversity suggests that immune systems are not defined by specific molecules, but by the design principles that can be implemented in multiple ways.

Innate immunity represents the most ancient instantiation of these principles. It relies on germline-encoded receptors that are immediately functional, evolutionarily stable, and biased toward conserved microbial features (1). By contrast, adaptive immunity in vertebrates introduces a radical shift in strategy: instead of encoding recognition space directly in the genome, it encodes a *process* that generates recognition diversity somatically (2). This shift trades genomic stability for flexibility and specificity, and is tightly coupled to increased organismal fitness, tissue and molecular complexity, and developmental investment (3, 4).

In jawed vertebrates, this strategy is realized through immunoglobulin (Ig)-superfamily antigen receptors—B-cell receptors and T-cell receptors (TCRs)—whose diversity is generated by V(D)J recombination. Recombination-activating gene 1 (RAG1) and RAG2 proteins, derived from a domesticated transposable element, catalyze site-specific DNA cleavage and rejoining at antigen-receptor loci (5–10). Combinatorial assembly of V, D, and J segments, together with junctional diversification mediated by error-prone DNA repair, generates a theoretical repertoire far exceeding what could be stably encoded in the germline (11). Loss or dysregulation of this system in humans leads to severe immunodeficiency, immune dysregulation, or malignancy, underscoring both its power and its inherent risk (12).

**Figure 1.**
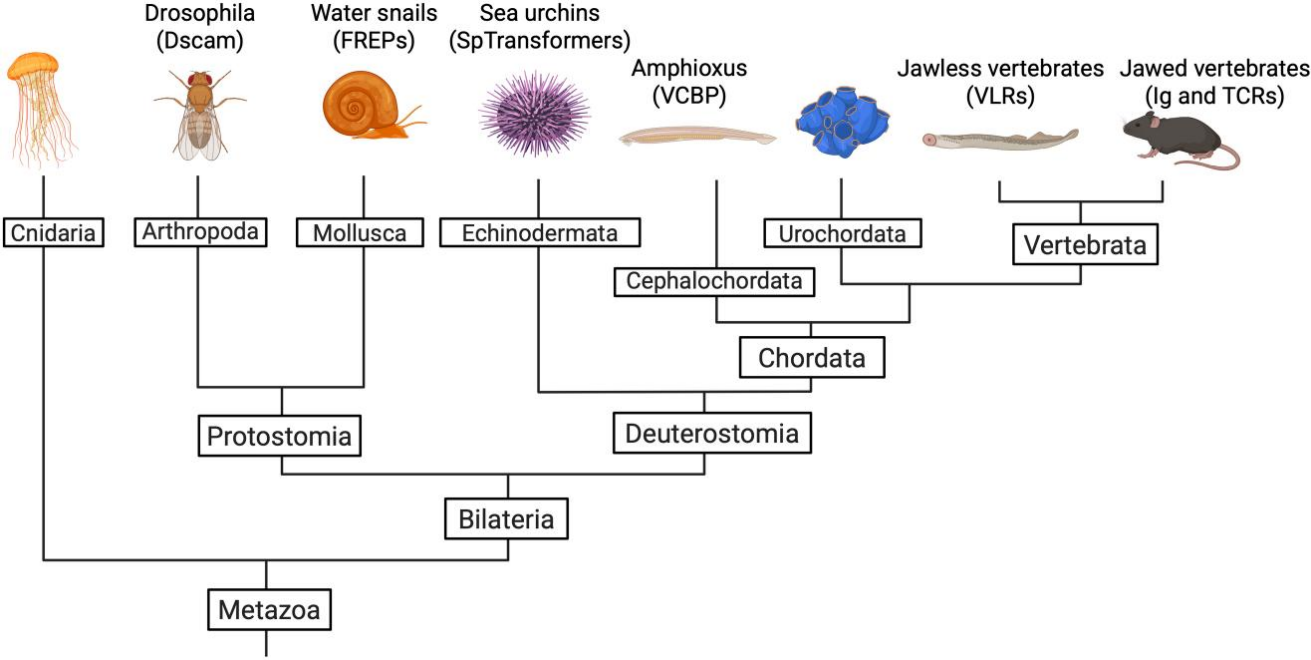
Evolutionary distribution of immune diversification strategies across metazoans. Simplified phylogeny of representative animal lineages highlighting distinct mechanisms of immune diversification. Protostomes employ strategies such as Dscam alternative splicing (arthropods) and FREP diversification (molluscs), whereas echinoderms rely on germline-encoded repertoires and coelomocyte-based responses. In chordates, cephalochordates use VCBPs, while vertebrates exhibit two forms of adaptive immunity: VLR-based systems in jawless vertebrates and Ig/TCR-based systems in jawed vertebrates. These examples illustrate multiple evolutionary solutions for generating immune recognition diversity.

Crucially, the absence of adaptive immunity does not imply immunological inferiority. Invertebrates and early diverging chordates thrive without lymphocyte-based adaptive immunity, often exhibiting sophisticated and dynamic innate defense strategies (Fig. 1). Adaptive immunity should therefore be viewed not as the apex of immune evolution, but as one of several viable solutions shaped by specific ecological and life-history constraints.

## Plasticity of adaptive immune architectures among vertebrates

Even within vertebrates, adaptive immunity is not monolithic. Comparative immunology reveals extensive variation in receptor structure, repertoire organization, and developmental timing. Cartilaginous fish possess unconventional Ig gene organizations and unique isotypes (13); marsupials exhibit delayed and environment-dependent immune maturation (14); camelids generate heavy-chain-only antibodies with distinct binding properties (15).

Jawless vertebrates, including lampreys and hagfish, present the most striking departure from the canonical model. Rather than Ig-based receptors, they employ variable lymphocyte receptors (VLRs) composed of leucine-rich repeat (LRR) modules (16, 17). These examples emphasize that adaptive immunity is defined less by molecular identity than by functional logic: somatic diversification, clonal expression, and selection.

The evolutionary tolerance for such diversity suggests that immune systems are constrained primarily by reproductive success rather than architectural optimality. As long as an organism can survive to reproduce, immune strategies can persist across evolution and thus vary dramatically. An extreme manifestation of this principle is observed in certain deep-sea anglerfish species, where males undergo sexual parasitism. Particularly, the species that show permanently fusion of multiple males to the female lose major components of adaptive immunity (18).

## Alternative adaptive immunity in jawless vertebrates: a mechanistic perspective

Jawless vertebrates possess a fully functional adaptive immune system that is mechanistically distinct yet conceptually convergent with that of jawed vertebrates. Their antigen receptors, VLRs, are assembled from arrays of LRR cassettes that are incomplete and non-functional in the germline configuration (16). During lymphocyte development, these cassettes are somatically assembled into mature VLR genes through a gene conversion-like process.

This process is mediated by cytidine deaminases of the AID/APOBEC (activation-induced cytidine deaminase/apolipoprotein B mRNA editing catalytic polypeptide-like) family (19), particularly CDA1 and CDA2, which introduce targeted DNA lesions that drive templated sequence insertion from surrounding donor cassettes (20–23 ). Distinct lymphocyte lineages expressing membrane-bound VLRA/VLRC and secreted VLRB emerge, exhibiting functional parallels to gnathostome T cells and B cells, respectively (24, 25). In addition to these three conventional VLRs, recent studies discovered more membrane-bound type VLRs (VLRD, E, and F), which suggested that they are expressed in T-like cells and function in a more cellular immunity context (26, 27).

Despite profound differences in molecular machinery, the jawless and jawed vertebrate systems share several core design elements: (i) antigen-receptor loci maintained in a non-functional germline state; (ii) lineage- and stage-restricted genome-editing enzymes; (iii) clonal expansion of lymphocytes expressing a single receptor specificity; and (iv) tight coupling of diversification to DNA-repair pathways. Notably, cytidine deaminases related to those used in jawless vertebrates are also employed in jawed vertebrates for somatic hypermutation and class-switch recombination of antibodies, reinforcing the idea of shared evolutionary toolkits (28, 29).

## Immune education and the necessity of tolerance

Somatic diversification of antigen receptors is a powerful but intrinsically dangerous strategy. Any process that generates recognition diversity at random will inevitably produce receptors with affinity for self-components. Adaptive immunity therefore cannot be understood solely as a mechanism for generating diversity; it must also include equally robust systems for tolerance implementation. From an evolutionary perspective, immune education is not an accessory feature but a co-equal requirement, without which somatic diversification would be incompatible with organismal viability or survival because of the possibility of autoimmune reactions.

In jawed vertebrates, this function is fulfilled by the thymus, a specialized epithelial organ that provides a structured microenvironment for T-cell development. Within the thymus, immature thymocytes undergo positive selection for self-MHC (major histocompatibility complex) recognition and negative selection to eliminate or divert cells bearing high-affinity receptors for self-antigens (30, 31). These processes are orchestrated by thymic epithelial cells, dendritic cells, and a network of transcriptional regulators, ultimately producing a repertoire that is both self-tolerant and responsive.

Jawless vertebrates lack gnathostome-type MHC molecules and TCRs, yet they face the same fundamental challenge. Lampreys possess a thymus-equivalent structure, the thymoid, located at the tips of the gill filaments (32). The thymoid supports the development of VLRA-expressing lymphocytes and exhibits spatial organization, proliferative zones, and gene expression patterns reminiscent of the vertebrate thymus. Developmentally, both organs arise from pharyngeal epithelium and depend on overlapping regulatory networks, most notably the transcription factor Foxn1, which acts as a master organizer of thymic epithelial identity (33, 34). This transcription factor emerged from ancestral Foxn4, which is also expressed in chordate pharyngeal epithelium and these two genes showed evolutionary trajectory of thymic function as a pan-lymphopoietic organ bipotent for both T lymphocytes and B lymphocytes to a highly T-cell-specialized educational microenvironment in jawed vertebrates (35–38).

The presence of a thymus-like education niche in jawless vertebrates strongly suggests that immune tolerance is not a late refinement but a foundational requirement for any adaptive immune system. Although the molecular mechanisms of selection remain incompletely understood in lampreys, available evidence supports the existence of selection processes that shape the VLR repertoire prior to peripheral deployment (39). Whether jawless vertebrates possess regulatory lymphocyte populations analogous to regulatory T cells, or rely on alternative peripheral tolerance mechanisms, remains an open question.

Taken together, these observations reinforce a central evolutionary principle: adaptive immunity requires the coordinated emergence of diversification mechanisms and tolerance-enforcing niches. Neither is viable in isolation.

## Diversification without lymphocytes: molecular strategies in invertebrates

**Figure 2.**
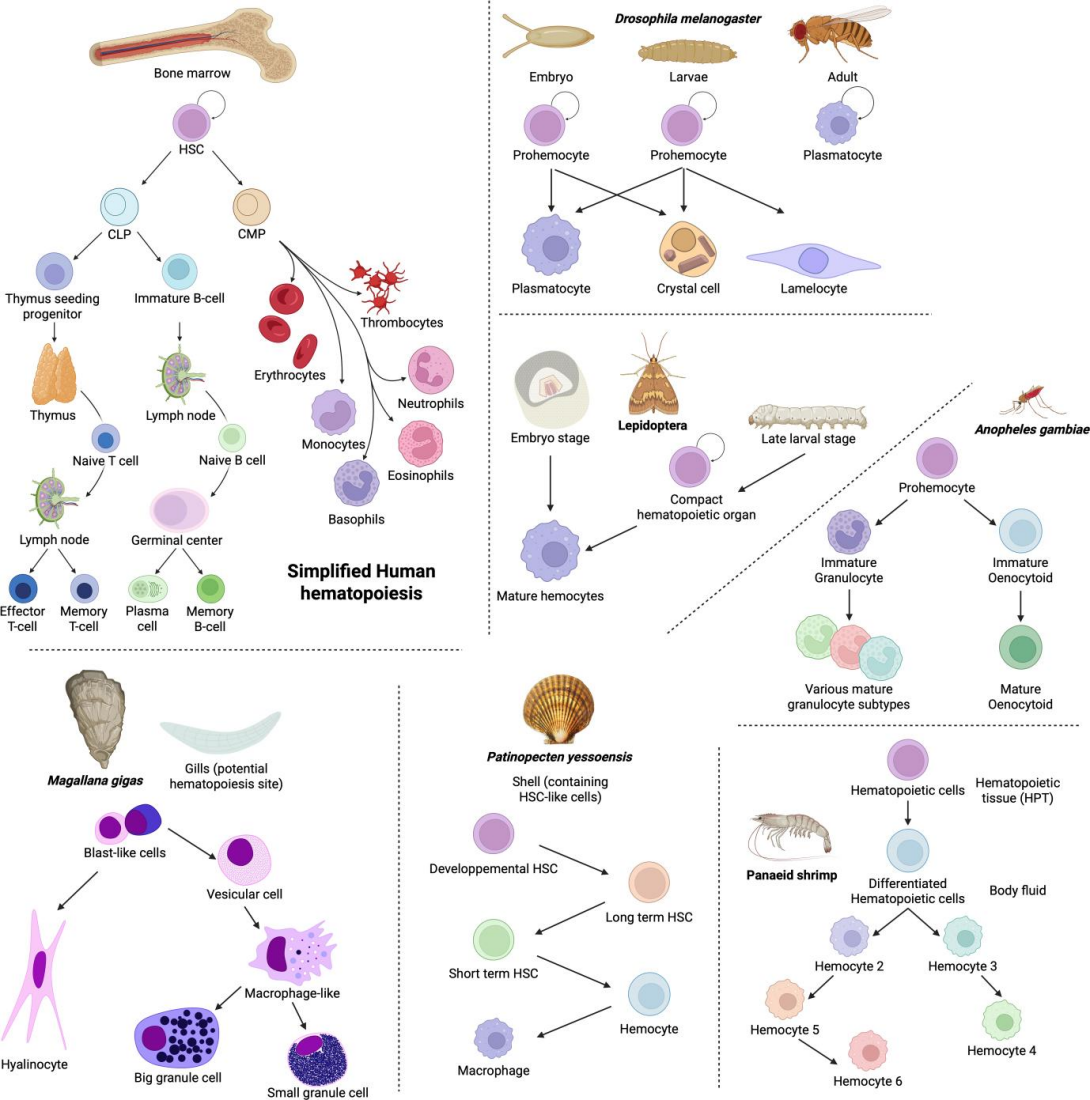
Contrasting architectures of immune diversification and cellular organization across Metazoa. This schematic compares representative strategies of immune diversification and their coupling to cellular differentiation and organization across major animal lineages discussed in this review. Vertebrates (upper left): in jawed vertebrates, adaptive immunity is built on somatic receptor diversification [V(D)J recombination] tightly coupled to proliferation-linked diversification. Multipotent hematopoietic stem and progenitor cells within structured niches (e.g. bone marrow, thymus) generate hierarchically organized lineages, including B and T lymphocytes that undergo clonal expansion and differentiate into effector and memory states. This architecture enables extreme receptor diversity but depends on centralized hematopoiesis, clonal selection, and regulated lineage commitment. Invertebrates: rather than requiring clonal proliferation and centralized control, many invertebrate systems implement distributed, non-clonal diversification strategies coupled to flexible cellular organization, thereby illustrating that immune systems represent multiple solutions within a shared design space across animal evolution.

Understanding the evolutionary origins of adaptive immunity requires stepping outside vertebrate-centric frameworks. Invertebrates dominate animal diversity and ecological space, and their immune systems represent long-standing, successful solutions to pathogen pressure. Rather than viewing invertebrate immunity as a primitive precursor, it is more productive to see it as an alternative evolutionary landscape in which many features associated with adaptive immunity, such as diversification, specificity, and inducibility, are achieved through different molecular strategies (Fig. 2).

Many invertebrates expand immune recognition capacity without clonal lymphocytes or heritable somatic recombination. Instead, they employ gene family expansion, allelic polymorphism, alternative splicing, and, in some cases, potential somatic sequence modification. These systems illuminate which aspects of adaptive immunity are contingent on lymphocytes and which reflect deeper, more general evolutionary design principles. Here we refer to several examples (also see Table 1).

**Table 1.** Immune diversification strategies across invertebrate lineages.

Taxon/model system	Diversification mechanism	Molecular basis	Functional context
Cephalochordates (amphioxus)	Population-level receptor diversity	VCBPs with Ig-like domains and chitin-binding modules; extensive allelic and copy-number variation	Gut-associated immunity; interactions with microbiota
Echinoderms (e.g. sea cucumbers, sea stars)	Population-level and cell-ensemble diversification	Large germline-encoded immune gene families; complement-like molecules; lectins	Coordinated coelomocyte responses, immune aggregation, and compartmentalized defense
Echinoderms (regeneration context)	Diversification through dynamic cell-state programs	Transcriptional reprogramming during tissue repair; immune–morphogenesis coupling	Regeneration-associated immune activation and tissue remodeling
Molluscs (e.g. *Biomphalaria*, bivalves)	Somatic and germline diversification of immune effectors	FREPs; gene duplication, allelic polymorphism, and somatic sequence diversification	Pathogen recognition and host–parasite interactions; dynamic expression following immune challenge
Arthropods (e.g. insects)	Alternative splicing-based receptor diversification	Down syndrome cell adhesion molecule (Dscam) generates tens of thousands of isoforms through mutually exclusive exon usage	Pathogen-specific immune responses in hemocytes

Cephalochordates such as amphioxus express variable-region-containing chitin-binding proteins (VCBPs), which combine Ig-like domains with chitin-binding regions and exhibit extensive allelic and copy number variation at the population level (40–42). VCBPs are expressed in gut-associated tissues and interact with commensal microbes, suggesting roles in immune–microbiota homeostasis in addition to direct pathogen elimination. Their population-level polymorphism provides diversity without somatic recombination.

In gastropods and bivalves, fibrinogen-related proteins (FREPs) constitute diversified immune effector families generated through gene duplication, allelic variation, and somatic sequence diversification (43). Individual animals can express distinct FREP repertoires, and expression profiles change dynamically following immune challenge.

The Down syndrome cell adhesion molecule (Dscam) in insects generates tens of thousands of isoforms via mutually exclusive alternative splicing (44–46). In *Drosophila* and mosquitoes, immune cells selectively express specific Dscam isoforms in response to pathogens, providing a form of molecular specificity without clonal selection.

Together, these examples reveal a progressive expansion of immune design space: germline polymorphism, inducible and partially somatic diversification, and transcript-level isoform complexity within individual cells. Echinoderms extend this trajectory further. Echinoderms occupy a uniquely informative position in comparative immunology. They share a deuterostome body plan with vertebrates yet lack lymphocyte-based adaptive immunity. This phylogenetic positioning allows an evolutionary test: which properties commonly associated with adaptive immunity require somatic recombination and clonal lymphocyte expansion, and which reflect more generalizable design principles? Rather than converging on a vertebrate-like solution, echinoderms elaborate immune complexity by expanding and dynamically deploying ancient innate toolkits. Recognition diversity is not generated through somatic genome rearrangement within individual cells; instead, it is distributed across large germline-encoded gene families, inducible transcriptional programs, and combinatorial effector ensembles. Echinoderms therefore exemplify an alternative immune architecture, one in which diversification and specialization arise without clonal receptor rearrangement.

## Germline expansion as a diversification strategy

A central feature of echinoderm immunity is the extensive expansion of pattern-recognition receptor repertoires, most thoroughly characterized in sea urchins. Genome annotation revealed extraordinary diversification of Toll-like receptors, NOD-like receptors, and scavenger-receptor-like genes (47, 48). Some receptor families encompass hundreds of loci, exhibiting lineage-specific expansions, polymorphism, and inducible expression dynamics. Unlike vertebrate antigen receptors, this diversity is germline-encoded but transcriptionally regulated. Immune challenge selectively upregulates distinct receptor subsets, reshaping recognition capacity at the cellular population level rather than within clonally expanded lineages. IL-17 pathway components and downstream inflammatory modules are particularly responsive (49), demonstrating that robust inflammatory logic can operate independently of somatic receptor recombination. Diversification is thus organized at the level of gene family architecture and regulated deployment rather than clonal genome editing (50). Recognition space is distributed across the genome and mobilized dynamically, illustrating that broad recognition capacity does not require rearranged receptors, but can emerge from expansion coupled to regulation.

## Effector diversification and structural plasticity

Echinoderms further amplify immune complexity through diversified effector families. The SpTransformer (Sp185/333) gene family in sea urchins provides a striking example (51, 52). This expanded locus encodes highly variable proteins generated through duplication, mosaic domain organization, and recombination-like restructuring within the cluster. Recombinant SpTransformer proteins display target-induced conformational flexibility and direct membrane interactions, including phosphatidic acid binding (53). Functional breadth appears to arise from structural adaptability rather than strict one-receptor–one-ligand specificity. Importantly, individual sea urchins differ in their expressed SpTransformer repertoires, and immune challenge reshapes the ensemble. Diversity is therefore manifested as a dynamic effector constellation rather than as fixed clonotypes. Specificity emerges from inducible combinatorial deployment rather than clonal selection, decoupling diversification from both somatic genome modification and lymphocyte proliferation.

## Cellular heterogeneity without clonal lineage commitment

Diversification extends to the cellular level. Coelomocytes comprise transcriptionally distinct functional states associated with phagocytosis, immune signaling, stress responses, and tissue interaction (54, 55). Rather than reflecting rigid lineage commitment, these subsets appear to represent context-dependent states within a shared cellular continuum. Anatomically localized immune-supporting tissues, discussed as hematopoietic or immune niches, sustain coelomocyte production and turnover (56). Although lacking organized lymphoid organs, these niches maintain functional specialization without lymphocyte-specific developmental programs. In vertebrates, the vast receptor diversity generated by V(D)J recombination necessitates intense proliferative clonal expansion. In echinoderms, diversification is encoded primarily through expanded germline families and inducible ensembles.

## Integration with homeostasis and regeneration

Sea stars illustrate how immune activation is embedded within organismal homeostasis. During sea star wasting-associated exposure, asymptomatic individuals sustain immune activation while preserving extracellular matrix programs (57). Effective immunity thus reflects coordinated defense and tissue maintenance rather than maximal pathogen elimination. Developmental and regenerative single-cell atlases provide frameworks for mapping immune programs across life stages (58), while chromosome-level assemblies enable analysis of gene family architecture (59). Immune activity in sea stars is integrated with structural stability and remodeling.

Sea cucumbers highlight an alternative organizational strategy in which immune functions are distributed across coelomocyte subtypes and coelomic compartments rather than being concentrated within a centralized hematopoietic organ. Defense responses are mediated by diverse coelomocytes together with soluble effectors present in the coelomic fluid. Transcriptomic analyses of cultured coelomocytes show robust induction of inflammatory and cytokine-like programs following stimulation with lipopolysaccharide (LPS), poly(I:C), or heat-inactivated *Vibrio* (60). Complement-related molecules, including C3-like and factor-B-like components, support opsonization and underscore a prominent fluid-phase arm of host defense (61, 62). Diversification is also expressed through coordinated immune-cell behavior. In holothurians, coelomocytes rapidly form aggregates whose cellular composition changes during maturation, consistent with stage-specific recruitment and remodeling (63). Such responses can be instructed by tissue-derived cues: in the sea cucumber *Apostichopus japonicus*, an intestine-derived galactose-binding C-type lectin (AjGBCL) accelerates coelomocyte migration and aggregate formation, suggesting that endogenous signals amplify collective responses during challenge (64).

Spatial organization further contributes to immune specialization. Distinct coelomocyte populations, including carotenoid-associated cells, are unevenly distributed across coelomic compartments such as the perivisceral fluid and hemal fluid associated with the Polian vesicles (65). Developmental timing also shapes immune organization: in the sea star *Patiria pectinifera*, allorecognition competence emerges after metamorphosis during the juvenile stage (66). These results indicate that diversification arises through compartmentalization and the partitioning of cellular and soluble effector activities among anatomical spaces, rather than through vertebrate-like centralized hematopoietic structures.

Regeneration provides an integrated perspective on this architecture, as tissue rebuilding intrinsically couples defense responses with morphogenesis. Single-cell RNA sequencing (scRNA-seq) profiling of regenerating holothurian intestine reveals flexible cell-state trajectories consistent with broad epithelial plasticity (67), while transcriptomic time-course analyses demonstrate coordinated dynamics between inflammatory activation and tissue remodeling programs (68, 69). These observations suggest that immune diversification and tissue repair represent coupled outputs of a shared injury-response network, in which host defense contributes not only to pathogen control but also to maintaining tissue integrity and supporting regeneration.

## Immune cell differentiation in invertebrates

As discussed above, the immune system structure is closely connected to the functional differentiation of cell lineages. However, the invertebrate hematopoiesis is not yet thoroughly understood. Invertebrate immune cells, commonly termed hemocytes or coelomocytes, have been classified according to morphology and staining properties. While cytology-based schemes remain operationally useful, they obscure functional heterogeneity and lineage relationships. Transcriptomic and single-cell approaches increasingly reveal that invertebrate immune systems comprise multiple immune cell states with specialized functions rather than a small number of static cell types. Recent single-cell analyses in oysters and other bivalves have identified distinct hemocyte populations defined by differential expression of genes associated with phagocytosis, pattern recognition, cytokine production, stress responses, and metabolic activity (70, 71). These findings suggest a previously underappreciated level of cellular organization and division of labor that parallels aspects of vertebrate immune compartmentalization (summarized in Table 2).

**Table 2.** Organization and differentiation of immune cell systems in invertebrates.

Taxon/system	Immune cell types	Differentiation/organization strategy	Evidence from recent studies	Functional implications
Echinoderms (sea cucumbers)	Multiple coelomocyte subtypes	Functional organization through spatial compartmentalization and collective behavior	Coelomocyte aggregation dynamics and immune activation studies	Coordinated immune responses across cell populations
Echinoderms (regeneration)	Plastic epithelial and immune cell states	Flexible cell-state trajectories during tissue repair	Single-cell profiling and transcriptomic time-course studies	Integration of immunity with regeneration programs
Sea stars	Coelomocytes with regulated allorecognition capacity	Developmental maturation of immune competence	Ontogenetic establishment of self–nonself recognition	Developmentally regulated immune discrimination
Bivalve molluscs	Hemocytes (granulocytes, hyalinocytes and additional transcriptionally defined subtypes)	Functional diversification revealed by single-cell transcriptomics	Distinct transcriptional programs associated with phagocytosis, signaling, and metabolism	Division of immune labor across hemocyte states
Gastropod molluscs	Hemocytes	Dynamic differentiation and activation states following infection	Transcriptomic and functional studies in host–parasite systems	Flexible immune effector responses
Arthropods	Hemocytes (plasmatocytes, crystal cells, lamellocytes in insects)	Developmentally specified lineages with infection-induced differentiation	Genetic and developmental studies in *Drosophila*	Specialized roles in phagocytosis, melanization, and encapsulation

Despite these advances, fundamental questions remain unresolved. The identity and hierarchy of hematopoietic progenitors, the presence and anatomical organization of immune niches (72), and the developmental trajectories linking hemocyte states are incompletely defined in most invertebrates (also see Fig. 2). Moreover, it remains unclear to what extent immune cell differentiation is developmentally hard-wired versus dynamically remodeled by environmental exposure and infection.

A central distinction between vertebrate and invertebrate immunity concerns the relationship between receptor diversification and cellular proliferation. In vertebrates, somatic recombination generates enormous antigen-receptor diversity, necessitating highly proliferative lymphocyte lineages capable of clonal expansion. Whether analogous proliferation-linked diversification operates in invertebrate immune lineages remains largely unexplored. Determining the proliferative capacity of hemocyte populations and how it scales with immune effector diversification, which is substantial but generally less extreme than vertebrate adaptive immunity, will be essential to clarify whether proliferation-driven repertoire maintenance represents a universal principle or a vertebrate-specific solution. Invertebrate hematopoiesis has been most intensively studied in a limited set of models spanning insects (e.g. *Drosophila melanogaster*), crustaceans of aquaculture relevance (shrimps and crabs), and molluscs with ecological, economic, or medical importance (bivalves such as *Magallana gigas* and gastropods such as *Biomphalaria glabrata*). Across Arthropoda and Mollusca, immune cell diversity emerges through lineage programs governed by defined developmental timing, plastically remodeled by microbial exposure, and maintained in adults by hematopoietic compartments of highly variable anatomical organization (Fig. 2).

In arthropods, hematopoiesis transitions from an active larval niche with spatial segregation of progenitor and differentiated zones to an adult system in which hematopoietic output is reduced and redistributed to peripheral compartments; adult maintenance relies largely on plasmatocytes, with proliferative capacity generally lower than during larval stages (73, 74). In *D. melanogaster*, hematopoiesis proceeds through two temporally and spatially organized waves. The embryonic wave arises in the head mesoderm and produces prohemocytes that differentiate into plasmatocytes and crystal cells, whereas a second wave in the larval lymph gland expands progenitors that generate plasmatocytes and crystal cells and can be redirected toward lamellocyte differentiation under immune challenge (75). Lineage commitment is governed by defined transcriptional programs: the GATA factor Serpent (Srp) together with Gcm regulates plasmatocyte differentiation; crystal cell differentiation depends on Runx family and Notch signaling. Hematopoiesis is dynamically remodeled by microbial and parasitic challenge. In parasitoid wasp infestation, lymph gland progenitors are redirected toward lamellocyte differentiation through Col/EBF-dependent transcriptional reprogramming and immune signaling pathways, notably JAK/STAT (74, 76).

Beyond *Drosophila*, insect hematopoiesis is anatomically and physiologically diverse. Multiple hematopoietic organs and proliferation of circulating or sessile hemocytes have been reported across species, varying with developmental stage, ecology, and infection history (75). In Lepidoptera, hemocyte proliferation depends on both embryonically derived hemocytes and compact hematopoietic organs that release mature hemocytes during late larval stages and degenerate around metamorphosis, indicating developmentally programmed niche reorganization (77, 78). In Orthoptera, a hematopoietic organ has been described, and hemocyte concentration increases after infection in *Locusta migratoria* (79). The persistence of inducible hematopoiesis in hemimetabolous insects could suggest that metamorphic remodeling in holometabolous species contributes to niche reorganization or reduction.

In mosquitoes, hematopoiesis and immune maturation are primarily characterized in adults, where physiology and infection alter hemocyte abundance and state. scRNA-seq identifies prohemocyte-like states ordered into two major lineages: an oenocytoid lineage dependent on lozenge expression and a granulocyte lineage progressing from immature to multiple mature subgroups (80). Circulating hemocytes can divide during infection, indicating that adult hematopoiesis may be supported by peripheral proliferation rather than exclusively by a discrete organ (81). Blood feeding induces transient hemocyte proliferation and activation via insulin signaling, with Ras–MAPK contributing to coupling feeding to immune activation (82–84).

In crustaceans, hemocytes derive from lobulated hematopoietic tissue containing progenitors at multiple differentiation stages. Hematopoiesis is regulated by extracellular signaling and tissue mechanics, including, transglutaminase-dependent extracellular matrix control, reactive oxygen species-associated regulation, and cytokines such as astakines. Classical morphology distinguishes hyaline, semigranular, and granular hemocytes, but molecular profiling indicates this scheme is method-dependent and insufficient to resolve lineage diversity (85). Terminal differentiation may continue after release into circulation, and the status of semigranular cells as obligatory intermediates remains debated (85–88). Single-cell studies in shrimp show infection-driven remodeling of hemocyte differentiation. Penaeid scRNA-seq identifies immature states and mature specialized states, with predicted trajectories. White spot syndrome virus infection reveals heterogeneous antiviral responses alongside inferred differentiation programs not yet causally linked to specific transcription factors (89–91).

In bivalves, early immune competence emerges during development, but hematopoietic trajectories generating adult hemocyte diversity remain less resolved than in insects. In *M. gigas*, immune gene expression is detected as early as gastrula–trochophore stages, preceding full circulatory establishment (92). scRNA-seq at the trochophore stage identifies hemocyte-like clusters expressing immune-associated genes with spatial localization by *in situ* hybridization, though lineage continuity to adult subsets remains unresolved (93). Later larval transcriptomics shows progressive upregulation of recognition, signaling, stress-response, apoptosis, and antimicrobial genes, consistent with maturation of effector capacity, but it remains unclear whether these patterns reflect differentiation trajectories, subset expansion, or activation remodeling (94). Microbial exposure during development reshapes Toll/NF-κB, JAK/STAT, and IFN-like pathways, with persistent juvenile phenotypes and enhanced resistance to ostreid herpesvirus type 1 (95). Developmental susceptibility to this virus underscores incomplete antiviral maturation in juveniles (96). Bacterial stimulation (*Vibrio splendidus*) induces hematopoiesis-associated transcription factors (GATA3, Runx) in gills (97, 98). Although dedicated hematopoietic organs remain debated, histology supports gills as candidate niches with Sox2-positive proliferative cells near hemolymphatic vessels (99). Across bivalves, proliferation of circulating hemocytes and tissue-associated progenitor compartments are both proposed (99, 100).

A major conceptual shift arises from evidence in *Patinopecten yessoensis* suggesting hematopoietic stem cell (HSC)-like cells in mineralized shell tissue, framing a bone-marrow-like niche (72). Whether these cells fulfill stem cell criteria (self-renewal, multilineage output, clonal persistence) remains unresolved. Hemocyte classification by morphology typically distinguishes granulocytes and hyalinocytes, whereas scRNA-seq reveals substantially richer diversity (71, 101, 102). In gastropods, classical morphology describes granulocytes and hyalinocytes, yet transcriptomics supports greater complexity. In water snail *B. glabrata*, scRNA-seq identifies at least seven hemocyte populations and suggests lineage or activation trajectories across multiple clusters (70). The anterior pericardial wall (APW, or amebocyte-producing organ) represents a putative hematopoietic site (103), with inducible mitotic activity after trematodes, LPS, fucoidan, and parasite antigens (104, 105), and transplantation experiments supporting hematopoietic function (106). Secondary hematopoiesis has also been proposed (107).

In cephalopods, the white body is a multilobed organ surrounding the optic tract and represents the primary hematopoietic site (108). Developmental staging describes hemocytoblasts differentiating into primary and secondary leukoblasts and then mature hemocytes (109). Circulating populations typically include dominant granular phagocytes and smaller, less granular cells (110). Transcriptomics of the white body in Euprymna tasmanica identifies hematopoiesis-associated genes (GATA2, FGFR2, MCL-1, TAF3, CD109), but integration into defined regulatory cascades remains incomplete (107, 111).

Across non-insect invertebrates, cell-state atlases often outpace causal inference. In molluscs and decapods, trajectory models are largely computational because transgenesis and lineage tracing are not routine, stem/progenitor criteria are rarely validated by clonal assays, and hematopoietic markers (e.g. Runx, GATA, Tal, Sox2) are frequently assigned by homology without functional validation. Consequently, claims such as ‘HSC-like cells’ require explicit definition of self-renewal and multilineage output. Evidence that infection redirects progenitor fate remains largely correlative. These limitations motivate cautious import of vertebrate single-cell conceptual frameworks, including continuum models of fate commitment, while recognizing that branching-versus-continuum architecture remains unresolved in most invertebrates (112).

## Conclusions

Comparative immunology reveals that immune systems are best understood not as linear progressions toward a single optimal solution, but as explorations of a shared design space. Adaptive immunity did not arise from scratch, nor did it emerge fully formed; rather, it represents a particular configuration of ancient molecular tools, including genome-editing enzymes, DNA repair pathways, transcriptional regulators, and developmental niches, assembled to meet the demands of vertebrate life histories. A central insight emerging from comparisons across Metazoa is the tight coupling between immune diversification and cellular organization (Table 3). In vertebrates, the extreme scale of receptor diversity generated by somatic recombination is inseparable from highly proliferative lymphocyte lineages and structured education niches that together sustain, select, and control vast repertoires. In contrast, invertebrate immune systems achieve functional diversity through molecular strategies that operate at more moderate scales and are not intrinsically linked to large-scale clonal proliferation. This contrast highlights proliferation-linked diversification as a lineage-specific solution rather than an inherent requirement for immune complexity.

**Table 3.** Evolutionary design principles of immune diversification: vertebrates vs invertebrates.

Feature	Vertebrate adaptive immunity	Invertebrate immune systems
Primary diversification mechanism	Somatic recombination of antigen-receptor genes [e.g. V(D)J recombination]	Germline gene family expansion, allelic polymorphism, alternative splicing, and somatic diversification of effector molecules
Cellular organization	Dedicated lymphocyte lineages (B cells and T cells)	Immune effector cells such as hemocytes or coelomocytes with multiple functional states
Repertoire generation	Extremely large receptor diversity generated within individual organisms	Diversity distributed across populations, gene families, and transcriptional programs
Clonal selection and expansion	Central principle: antigen-specific lymphocytes proliferate upon activation	Generally absent; immune responses often rely on coordinated activity of heterogeneous cell populations
Immune memory	Long-lived antigen-specific memory cells	Limited or context-dependent memory-like phenomena reported in some systems
Spatial organization	Centralized hematopoietic organs (bone marrow, thymus) and secondary lymphoid tissues	Often decentralized immune architecture with immune cells distributed in body cavities or tissues
Diversification–proliferation relationship	Extensive receptor diversification coupled to strong proliferative capacity of lymphocyte lineages	Diversification typically occurs without large-scale clonal proliferation

Invertebrates and jawless vertebrates thus demonstrate that many features often considered hallmarks of jawed vertebrate adaptive immunity can be implemented through alternative molecular and cellular routes. As emerging technologies make non-conventional model organisms increasingly accessible, these systems offer unique opportunities to dissect which aspects of immune design are necessary, which are contingent, and which are repeatedly reused. Beyond comparison, they also provide conceptual insight: alternative strategies for molecular diversification, cellular plasticity, and distributed immune organization may inform future efforts in biotechnology and medicine, including immune engineering and genome editing. At the same time, a better understanding of why vertebrates evolved highly proliferative, clonal immune systems may help clarify the constraints that shape immune system function and dysregulation. Studying these systems is therefore not merely comparative; it is foundational to understanding what immunity is.

Looking forward, several key questions will shape the next phase of evolutionary immunology. How distinct immune architectures balance diversification, specificity, and regulation without converging on lymphocyte-based clonal expansion remains a central challenge. In particular, the relationship between receptor diversification and cellular organization is unresolved: to what extent are large-scale diversification strategies inherently linked to proliferative lineages, and can comparable functional complexity emerge from distributed, non-clonal systems? Addressing these questions will require integrating single-cell approaches with functional and lineage-tracing frameworks across diverse taxa. In parallel, it remains unclear how deeply immune systems are embedded within broader programs of tissue homeostasis, regeneration, and host–microbiota interactions. Finally, understanding how developmental timing, environmental exposure, and evolutionary history shape immune system design will be essential for identifying general principles beyond vertebrate-centric models. Resolving these challenges will not only refine our understanding of immune evolution, but also reframe immunity itself as a diverse and evolving set of solutions to a common biological problem.
